# Rearranged Copolyurea Networks for Selective Carbon Dioxide Adsorption at Room Temperature

**DOI:** 10.3390/polym13224004

**Published:** 2021-11-19

**Authors:** Junsik Nam, Eunkyung Jeon, Su-Young Moon, Ji-Woong Park

**Affiliations:** 1 School of Materials Science and Engineering, Gwangju Institute of Science and Technology, 123 Cheomdangwagi-ro, Buk-gu, Gwangju 61005, Korea; namjunsik@gist.ac.kr (J.N.); jeon8577@gmail.com (E.J.); 2 Carbon Resources Institute, Korea Research Institute of Chemical Technology, 141 Gajeongro, Yuseong, Daejeon 34114, Korea; msy1609@krict.re.kr

**Keywords:** urea network polymer, copolymer, porous polymer, carbon dioxide adsorbent, carbon dioxide selectivity

## Abstract

Copolyurea networks (co-UNs) were synthesized via crosslinking polymerization of a mixture of tetrakis(4-aminophenyl)methane (TAPM) and melamine with hexamethylene diisocyanate (HDI) using the organic sol-gel polymerization method. The subsequent thermal treatment of between 200 and 400 °C induced the sintering of the powdery polyurea networks to form porous frameworks via urea bond rearrangement and the removal of volatile hexamethylene moieties. Incorporating melamine into the networks resulted in a higher nitrogen content and micropore ratio, whereas the overall porosity decreased with the melamine composition. The rearranged network composed of the tetraamine/melamine units in an 80:20 ratio showed the highest carbon dioxide adsorption quantity at room temperature. The results show that optimizing the chemical structure and porosity of polyurea-based networks can lead to carbon dioxide adsorbents working at elevated temperatures.

## 1. Introduction

Porous organic networks have been studied for use as gas adsorbents [1,2,3,4,5,6,7], owing to their high surface area and microporosity [8,9], the tuneability of their organic chemical structures and microstructures for high adsorption efficiency, and selectivity to a targeted gas [1,10,11,12]. Capturing carbon dioxide (CO_2_) using organic porous materials has drawn particular attention [13,14], yielding many adsorbents that exhibit high adsorption quantity with selectivity. However, the gas adsorption performances of most CO_2_ adsorbents have been measured at 273 K or lower temperatures. They usually exhibited poor adsorption efficiency and selectivity, even at room temperature, limiting the practical application of the materials for capturing CO_2_ without the additional energy penalty of cooling. Therefore, a study on a CO_2_ adsorbent with high adsorption efficiency and selectivity at a higher temperature is demanded. Different CO_2_ adsorbents that can be operated above room temperature have been developed [15,16].

At least two main characteristics of the organic porous materials have been identified as effective for improving CO_2_ adsorption performance: the microporosity and heteroatom composition. For the first, increasing the number of micropores in the adsorbent provides a way to enhance the CO_2_ adsorption selectivity. The kinetic size of CO_2_ is known to be 330 pm, smaller than that of nitrogen, oxygen, argon, and methane, which are the other components of general CO_2_-containing mixed gases [17,18]. Therefore, CO_2_ molecules are adsorbed preferentially to the smaller pores by the molecular sieving effect. In the second, introducing heteroatoms such as nitrogen into the porous structure increases the adsorption selectivity. The nitrogen atom has a partial negative charge due to its lone pair electrons and its high electronegativity [19,20]. Such a Lewis base character provides a chemical affinity toward CO_2_. Organic porous materials with structures easily controllable in terms of the microporosity and the heteroatomic content are suitable candidates for adsorbing CO_2_ at higher temperatures.

Polyurea networks, or urea-bonded porous networks (UNs), have shown the ability to be rearranged into hierarchical porous frameworks that selectively adsorb CO_2_ [21]. UNs can be synthesized by a solution polymerization employing the organic sol–gel method, analogously to the sol–gel synthesis developed mainly for inorganic network materials [22,23,24]. A typical system is a reaction between a tetra-functional aromatic amine and an alkyl diisocyanate in dimethylformamide [25,26,27]. Below the critical gelation concentration or before the gelation time, the polymerization yields nanogels, i.e., nanoparticulate networks, which are well-dispersed in the solution. Precipitating the UN nanogels into a nonsolvent provides porous UNs, which can be transformed into rearranged urea-bonded networks (RUNs), consisting of hierarchically porous organic frameworks via thermal rearrangement and the removal of volatiles upon heating above 200 °C [21,25].

RUNs show a considerable CO_2_ adsorption quantity, with a high selectivity even at room temperature, which appeared comparable to or better than many other materials reported to date [21]. Since a UN can be synthesized from many different amines and isocyanate monomers [25,28], RUNs’ CO_2_ adsorption performance may be improved further by optimizing their chemical structure for the micropore content and heteroatom composition.

The unique micropore generation process of a UN starts from the thermal cleavage of urea bonds between the nonvolatile aromatic and volatile aliphatic moieties with amine or isocyanate functional groups and the concurrent or subsequent cyclization of the isocyanates of the residual structure into isocyanurate rings [21]. The expulsion of volatile aliphatic moieties and the irreversible formation of isocyanurate nodes results in the transformation of a UN into a microporous framework. From the understanding of the mechanism, we anticipated that increasing the weight fraction of the aliphatic diisocyanate monomer would provide a UN with a high aliphatic ratio, which could result in an RUN with a higher microporosity upon thermal treatment. In addition, the residual microporous framework resulting from thermal rearrangement needs as high a nitrogen content as possible to enhance the affinity to carbon dioxide.

In our previous report, the amine/isocyanate monomer pair used for the synthesis of an RUN was tetrakis(4-aminophenyl)methane (TAPM) and hexamethylene diisocyanate (HDI) [21]. The weight loss that occurred upon thermal treatment was close to the ratio of HDI in the monomer mixture. To increase the weight fraction of an aliphatic moiety, one must use aromatic amines with a smaller molar mass than TAPM. In the meantime, it would be better for the residual aromatic units to contain more nitrogen atoms in their structure. These considerations led us to choose melamine as the amine monomer to obtain high-microporosity, nitrogen-rich RUNs. Melamine consists of a tri-amino pseudo-aromatic ring with a molar mass of about one-third of the TAPM, containing six nitrogens among 15 non-hydrogen elements per molecule. Nevertheless, it is still necessary to study the chemical reactivity of the melamine molecules for the organic sol–gel polymerization, in addition to the effect of the chemical and geometrical structure of the flat melamine units on the thermal rearrangement and micropore generation of the resultant polyurea network.

Here, we synthesized copolyurea networks by polymerizing a mixture of TAPM and melamine with HDI through the organic sol–gel method, and studied the effect of the melamine composition on the porosity and CO_2_ adsorptivity of the resultant thermally rearranged microporous network. The melamine-added UN transformed into a rearranged copolyurea network consisting of a nitrogen-rich micropore. Further optimization of the melamine composition in the precursor network resulted in an RUN with a large specific surface area with the highest CO_2_ adsorption selectivity at room temperature.

## 2. Materials and Methods

### 2.1. Materials

Tetrakis(4-aminophenyl)methane (TAPM) was prepared by the previously reported method [29]. Hexamethylene diisocyanate (HDI) (99%, Sigma-Aldrich, St. Louis, USA) was freshly distilled under reduced pressure. *N*, *N*-dimethyl formamide (DMF) (anhydrous, 99.8%, Sigma-Aldrich, St. Louis, MO, USA), dimethyl sulfoxide (DMSO) (anhydrous, 99.9%, Sigma-Aldrich, St. Louis, MO, USA), and melamine (99%, Sigma-Aldrich, St. Louis, USA) were used without further purification.

### 2.2. Preparation of Rearranged Copolyurea Networks (co-RUNs)

Copolyurea networks (co-UNs) were prepared with six different mole ratios (TAPM: melamine = 100:0, 99:1, 80:20, 60:40, 40:60, and 0:100); each co-UN was denoted as T100, T99M1, T80M20, T60M40, T40M60, and M100, respectively. In a typical run for the synthesis of T80M20, TAPM (0.82 mmol, 0.31 g) was dissolved in DMF (7.84 mL), the melamine (0.21 mmol, 0.026 g) was dissolved in DMSO (0.65 mL), the HDI (1.96 mmol, 0.33 g) was dissolved in DMF (8.23 mL), the TAPM solution was added into the HDI solution, and then the melamine solution was added to the resulting TAPM/HDI mixture drop-wise at room temperature under a nitrogen atmosphere. The mixture was stirred until it became gel. The gelled mixture was precipitated into a copious amount of deionized water. The precipitate was washed three times with acetone. The powdery solid was isolated by filtration and dried for 48 h at 150 °C in a vacuum oven (VOS-310C, Sunil Eyela, Sungnam, Korea). T100, T99M1, T80M20, T60M40, T40M60, and M100 were prepared using the same procedure, except for the volumes of TAPM and melamine solutions. The as-prepared co-UN samples were thermally treated in a programmable muffle furnace (Daihan FX-27, Daihan Scientific, Wonju, Korea), yielding the corresponding co-RUN. The samples were heated to 380 °C at a rate of 2 °C/min under the nitrogen atmosphere, kept for 1 h at the final temperature, and then cooled rapidly to room temperature. The resulting co-RUNs were denoted as R-T100, R-T99M1, R-T80M20, R-T60M40, R-T40M60, and R-M100.

## 3. Results and Discussions

**Figure 1:**
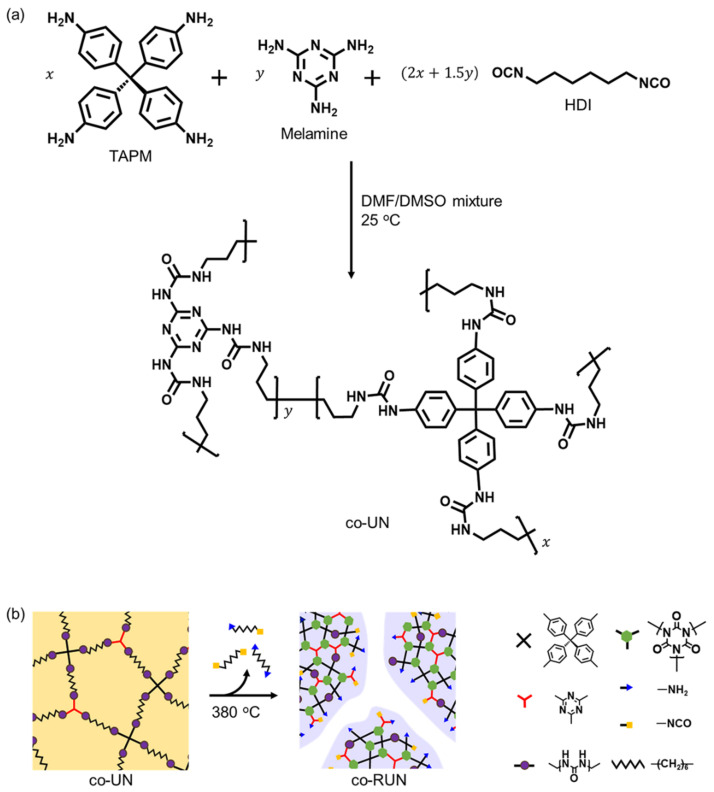
Synthesis of a copolyurea network (co-UN) and its thermal transformation into a rearranged copolyurea network (co-RUN). (**a**) Polymerization of TAPM, melamine, and HDI to obtain a co-UN. (**b**) Schematic illustration of the thermal rearrangement of a co-UN into a microporous co-RUN.

Copolyurea networks were prepared by reacting TAPM (T), melamine (M), and HDI in a mixed solvent of DMF and DMSO (Figure 1a). The products were isolated as powdery solids by precipitation of the gelled mixtures into water. We prepared six different co-UNs with TAPM to melamine molar ratios of 100:0, 99:1, 80:20, 60:40, 40:60, and 0:100. The detailed compositions of TAPM, melamine, and HDI are listed in Table S1. The resultant co-UNs were denoted as T100, T99M1, T80M20, T60M40, T40M60, and M100.

In synthesizing the co-UNs, a mixed solvent of DMSO and DMF was used because of the low solubility of melamine in DMF. The melamine was dissolved in DMSO, and the TAPM and HDI were dissolved in DMF. The melamine solution was added slowly to the reaction mixture of TAPM and HDI. The final reaction mixture maintained a transparent sol state, not forming precipitation until it became a gel. The homo-polyurea network of TAPM and HDI (denoted as T100) became a gel after 72 h of reaction in DMF [25], whereas that of melamine and HDI (M100) in DMSO was gelled after 103 h. Only a slight difference in the gelation time suggested that the TAPM and melamine could be copolymerized in the mixed solvent system. T99M1, T80M20, T60M40, and T40M60 in a solvent of DMF and DMSO mixed proportionally to the T/M ratio were gelled after 72, 79, 87, and 91 h, respectively. The increase in gelation time with the melamine ratio within the range of 72 to 100 h indirectly indicated the formation of co-UNs with the intended composition.

The as-prepared co-UN samples were heated slowly to the final temperature and kept for 1 h to be transformed into their respective co-RUNs. The thermal rearrangement temperature was optimized to obtain co-UNs with a high CO_2_ adsorptivity. For T80M20, its rearranged product showed the highest CO_2_ adsorption quantity when heated at 380 °C (Figure S1). The other co-UNs were treated using the same heating conditions to obtain corresponding co-RUNs.

The transformation of a co-UN into a co-RUN is schematically shown in Figure 1b, which should be identical to the mechanism generating the microporous structures known for the homo-polyurea network [21]: the urea bonds of the co-UN start to dissociate to generate amine and isocyanate groups above 200 °C. During this process, alkyl fragments are released and vaporized to leave micropores. The residual network rearranges to a microporous isocyanurate framework.

**Figure 2:**
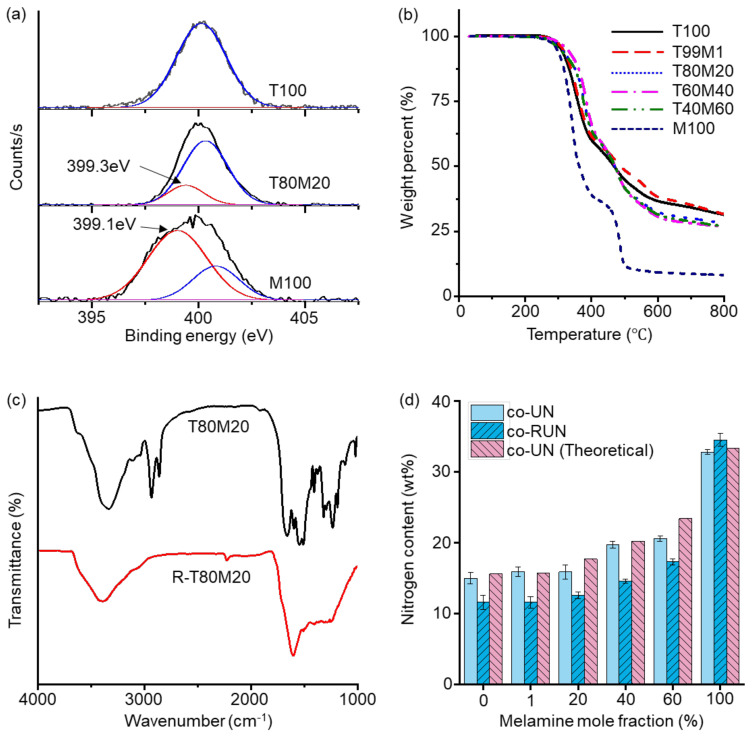
The transformation of the chemical structure from co-UNs into co-RUNs. (**a**) XPS N1s spectra of T100, T80M20, and M100 (red: triazine, blue: urea bond). (**b**) TGA curve of co-UNs. (**c**) FT-IR spectrum of T80M20 and R-T80M20. (**d**) Nitrogen content of co-UNs and co-RUNs, respectively, plotted against the molar ratio of the melamine in the amine monomer mixture.

The inclusion of melamine into the co-UNs was confirmed by X-ray photoelectron spectroscopy (XPS). The T100, consisting only of the TAPM/HDI network, showed only a peak of N1s at 400.1 eV, corresponding to the urea bond, whereas T99M1, T80M20, T60M40, T40M60, and M100 exhibited an additional N1s peak at 399.3 eV, corresponding to triazine (Figure 2a and Figure S2) [30]. The increasing intensity of the triazine N1s peak confirmed that the TAPM and melamine were copolymerized successfully with HDI, according to the monomer ratios (Table S2).

Thermogravimetric analyses (TGAs) and Fourier-transform infrared (FT-IR) spectra confirmed the rearrangement of the co-UNs into the co-RUNs. The initial decomposition of the co-UNs took place between 300 and 400 °C (Figure 2b), similar to the homo-polyurea network reported previously [21]. The evaporation of the hexamethylene moiety was the main cause of the weight loss during the rearrangement, as indicated by the disappearance of the C–H stretching band in 2930 and 2860 cm^−1^. The peak near 1710 cm^−1^ in the FT-IR confirmed the generation of the isocyanurate ring (Figure 2c and Figure S3).

The elemental analysis of the co-UNs and co-RUNs confirmed the increase in nitrogen content with their melamine ratios. The nitrogen weight fraction increased from 15 to 33 wt% in the co-UNs and 12 to 35 wt% in the co-RUNs as the melamine ratio in the amine monomer was varied from 0% to 100% (Figure 2d, Table S3). The nitrogen fractions in the co-UNs were nearly identical to their theoretically estimated values, indicating that the melamine ratio successfully controlled the incorporation of nitrogen.

TGA of co-UNs was performed to see how the thermogravimetric change is dependent on the ratio of monomers incurred by the addition of melamine. The co-UNs decomposed via two-stage weight loss (Figure 2b). The first weight loss occurred below 400 °C with the evaporation of alkyl chains in the range of urea bond thermolysis and a rearrangement reaction [21]. The second weight loss proceeded above 400 °C with the carbonization of organic matter [31]. The first-stage weight losses of T100 and M100 were about 40 and 65%, respectively. In the co-UNs, the separation of the first- and second-stage weight losses was less visible. Nevertheless, the weight loss of the co-UNs at 800 °C increased with the weight percentage of the aliphatic moiety (Table S4).

**Figure 3:**
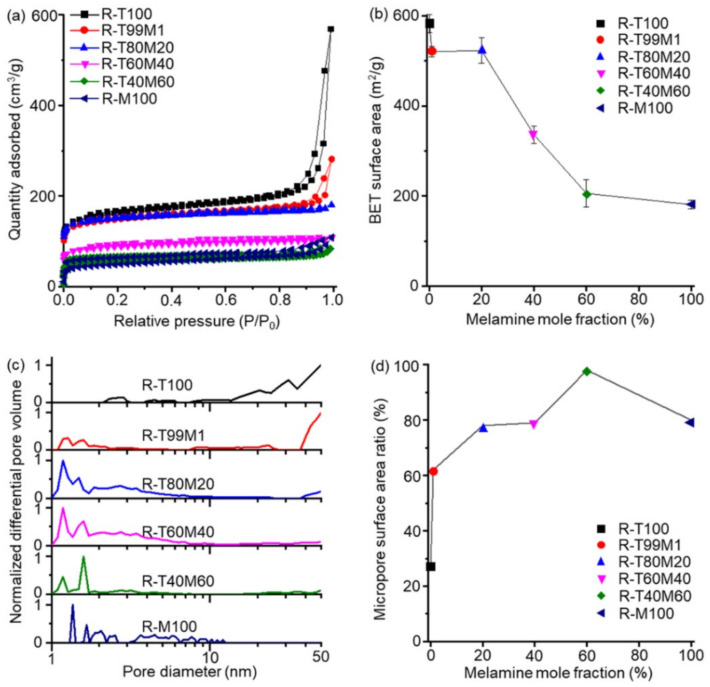
Gas adsorption–desorption isotherm experiments and pore distribution of co-RUNs. (**a**) N_2_ adsorption–desorption isotherm curves were collected at 77 K for co-RUNs. (**b**) The BET surface area of co-RUNs. (**c**) The normalized NLDFT pore distribution curve between 1 nm and 50 nm of co-RUNs. The curves were derived from the N_2_ adsorption isotherm data collected at 77 K using the N_2_ cylindrical porous carbon model. (**d**) The ratio of S_micro_ over S_total_ of co-RUNs.

The effect of the melamine content on the co-RUNs porosity was drawn from the isothermal N_2_ adsorption–desorption data (Figure 3a). The adsorption amount of gas adsorbed to the co-RUNs was reduced at higher melamine ratios, giving specific surface areas correspondingly. The BET surface area was 583 m^2^/g for R-T100 and slightly decreased to 521 m^2^/g and 523 m^2^/g for R-T99M1 and R-T80M20, respectively (Figure 3b, Table S5). The surface areas dropped to 336, 206, and 181 m^2^/g for R-T60M40, R-T40M60, and R-M100. The adsorption isotherms showed that the reduction in the adsorbed amount of gas was particularly prominent in the region over 0.8 P/P_0_ at a higher melamine ratio, indicating that the addition of melamine units prevented macropore formation. The SEM of the co-RUNs showed that the macroporous structure collapsed at a high melamine ratio (Figure S4). This result is most likely caused by the high fraction of the flexible alkyl moiety in the high-melamine co-UNs, causing the structure to collapse into a less porous structure during the thermal cleavage and rearrangement process.

Although incorporating melamine units decreased the overall pore volume, it changed the pore distribution to allow for more selective adsorption of CO_2_. The volume of smaller pores (micro- or meso-) in the co-RUNs was estimated from the normalized NLDFT pore distribution curves, which clearly showed that the addition of melamine significantly reduced the ratio of mesopores, whereas it increased the ratio of micropores in the co-RUNs (Figure 3c). As a result, the ratio of the micropore surface area (S_micro_) of the pores smaller than 2 nm to the total surface area (S_total_) of the pores smaller than 50 nm increased from 30% to over 90% with an increase in the melamine ratio (Figure 3d).

**Figure 4:**
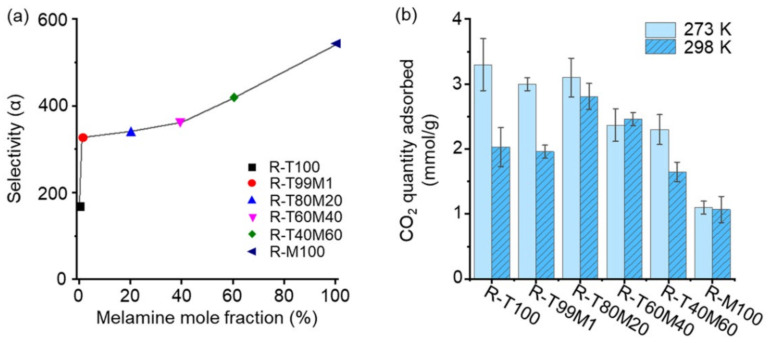
CO_2_ adsorptivity of co-RUNs. (**a**) The IAST adsorption selectivity at 1 bar. (**b**) CO_2_ adsorption quantity at 1 bar for co-RUNs collected at 273 K and 298 K.

As expected, the high micropore ratio and nitrogen content of the co-RUNs with melamine units led to high CO_2_ adsorption selectivity. The CO_2_ adsorption selectivity estimated using the ideal adsorbed solution theory (IAST) model from the N_2_ and CO_2_ isothermal adsorption data appeared to increase with the molar ratio of melamine in the amine monomers (Figure 4a, Figures S5 and S6).

The heat of adsorption values of co-RUNs, influencing the adsorption selectivity, was calculated by using the CO_2_ adsorption data collected at 273 K (Figure S7) and 298 K (Figure S5a). It is noticeable that the heat of adsorption decreased with the amount of incorporated melamine (Figure S8). The result may be explained by the lower basicity of amino groups of the melamine (pK_b_ = 9.0) [32] than those of the TAPM (similar to aniline, pK_b_ ~ 9.4) [33]. Thermal treatment will remove aliphatic moieties and generate pores. The pores of the resultant rearranged networks are enriched with aromatic isocyanurate and urea groups on their surfaces. The urea or isocyanurate bonds of less-basic amino groups of melamine may show a weaker affinity to carbon dioxide.

The significant increase in CO_2_ adsorption selectivity with the addition of melamine resulted in different CO_2_ adsorption performances in response to an increase in temperature (Figure 4b and Figure S7). The CO_2_ adsorption quantities measured at 273 K for R-T100, R-T99M1, and R-T80M20 were 3 mmol/g or higher and decreased significantly for R-T60M40, R-T40M60, and R-M100, following the same trend as the BET surface area. By contrast, a different result was obtained when the CO_2_ adsorption quantities of all co-RUNs were measured at 298 K (Figure 4b and Figure S5a). The CO_2_ adsorption quantity at 298 K for R-T80M20 and R-T60M40 with a melamine composition of 20 and 40%, respectively, appeared higher than that for R-T100 and R-T99M1. R-T80M20 showed the highest adsorption amount at 298 K, whereas R-T100 containing no melamine unit did at 273 K. The quantity adsorbed by R-T100 at 298 K was only 60% of that at 273 K. We assume that the high CO_2_ adsorption quantity of R-T80M20 at room temperature is owing to its high CO_2_ selectivity and surface area. In the case of R-T60M40, R-T40M60, and R-M100, although they have high selectivity, their surface areas and thus pore volumes were too low to adsorb the gas in a sufficient amount.

## 4. Conclusions

In summary, incorporating melamine into urea-based networks resulted in higher nitrogen contents and aliphatic weight fractions, providing rearranged copolyurea networks with higher micropore ratios and nitrogen contents upon thermal treatment. Furthermore, the rearranged copolyurea networks showed higher CO_2_ adsorption selectivity than the previously reported RUNs. In particular, the RUNs composed of the melamine/tetraamine units in a 20:80 ratio showed the highest CO_2_ adsorption quantity at room temperature. The results show that the relative weight fraction of the aliphatic moiety in the UNs can be increased using an amine co-monomer with a smaller molecular weight and higher nitrogen content. Since the micropores of the UNs are generated upon thermal treatment via the loss of aliphatic units, co-UNs with a higher melamine content gave the rearranged UNs an increased microporosity, allowing for a higher CO_2_ adsorption selectivity, which exhibited a higher CO_2_ adsorption quantity at room temperature than the previously reported RUNs. The result also shows that optimizing the chemical structure and porosity of polyurea-based networks by adjusting the ratio of different aromatic amine co-monomers that are polymerized with an alkyl diisocyanate can lead to more selective carbon dioxide adsorbents working at elevated temperatures. It emphasizes that the performance of the CO_2_ adsorbent should be maximized in the microporosity and heteroatomic content while increasing the total surface area.

## Supplementary Materials

The followings are available online at https://www.mdpi.com/article/10.3390/polym13224004/s1, Figure S1: CO_2_ adsorption quantity depending on the final heating temperature, Figure S2: XPS N1s spectrum of co-UNs, Figure S3: FT-IR spectra of co-UNs and co-RUNs, Figure S4: SEM images of co-RUNs, Figure S5: N_2_ and CO_2_ adsorption isotherms of co-RUNs collected at 298 K, Figure S6: CO_2_ IAST selectivity of co-RUNs, Figure S7: CO_2_ adsorption-desorption isotherm curve of co-RUNs, Figure S8: CO_2_ heat of adsorption of co-RUNs, Table S1: Monomer composition of co-UNs, Table S2: Nitrogen functional group ratio of co-UNs, Table S3: Elemental ratio of co-UNs and co-RUNs, Table S4: Estimated monomer weight percent of co-UNs, Table S5: BET surface area and t-plot micropore surface area of co-RUNs.
