# Supplementary material for: Rearranged Copolyurea Networks for Selective Carbon Dioxide Adsorption at Room Temperature
Source: Polymers (Basel). 2021 Nov 19;13(22):4004. doi: 10.3390/polym13224004 (PMC8623474; doi:10.3390/polym13224004)
Supplement: Supplementary file 1 [file polymers-13-04004-s001.zip › polymers-1443316-supplementary.pdf]

Supplementary Information

Rearranged copolyurea network for selective carbon dioxide adsorption at room temperature

Junsik Nam,[1] Eunkyung Jeon,[1] Su-Young Moon,[2] and Ji-Woong Park[1,*]

[1] School of Materials Science and Engineering, Gwangju Institute of Science and Technology, 123 Cheomdangwagi-ro, Buk-gu, Gwangju 61005, Korea
[2] Korea Research Institute of Chemical Technology, Carbon Resources Institute, 141 Gajeongro, Yuseong, Daejeon, 34114, Korea

Corresponding author: Ji-Woong Park

School of Materials Science and Engineering, Gwangju Institute of Science and Technology,

123 Cheomdangwagi-ro, Buk-gu, Gwangju 61005, Korea

Telephone: +82-62-715-2315

Fax: +82-62-715-2304

E-mail: jiwoong@gist.ac.kr.

Experimental

Characterizations

Scanning electron microscopy (SEM)

SEM images were collected on high-resolution scanning electron microscopy (HR-SEM, JSM-6700F, JEOL). Before taking SEM measurements, samples were dried under a vacuum for 24 hours and coated with platinum by sputtering for 60 seconds.

X-ray photoelectron spectroscopy (XPS)

The chemical structure was evaluated using an X-ray photoelectron spectrometer (Thermo Fisher Scientific, VG Multilab 2000) equipped with an Al K-Alpha energy source under ultrahigh vacuum conditions. Scan number is 3. The energy step size is 1.0 eV. For sample preparation, the powder samples were packed in a 1 cm diameter of the sample holder.

Elemental analysis

The elemental analysis was analyzed by EA2000 and EA1112 (Termofinnigan). Elemental analysis was conducted at 1000 °C. Tungstic anhydride, copper wire, nickel-plated carbon, and quartz turnings were used as a catalyst.

Gas adsorption-desorption isotherm experiment

Gas adsorption-desorption isotherms were performed with an ASAP 2020 volumetric adsorption apparatus (Micromeritics, USA). Before analysis, the samples were degassed in the degassing port of the adsorption analyzer at 423 K for at least 12 hours. N_2 adsorption-desorption isotherm curves were collected at 77 K and 298 K. CO_2 adsorption-desorption isotherm curves were collected at 273 K and 298 K. The specific surface area was calculated with the Brunauer-Emmett-Teller (BET) theory by using the N_2 isotherm adsorption curve. The micropore distribution curve was calculated with nonlocal density functional theory (NLDFT) by using CO_2 isotherm adsorption data. The heat of adsorption for carbon dioxide was calculated from the adsorption isotherms collected at 273 K and 298 K by using ASAP 2020 v3.00 software.

Fourier transform infrared (FT-IR) spectroscopy

Fourier transform infrared (FT-IR) spectroscopy was carried out on a Thermo Fisher Scientific Nicolet iS10 FT-IR Spectrometer. Powder samples for FT-IR were prepared in KBr pellets.

Thermogravimetric analysis (TGA)

Thermogravimetric analysis (TGA) of the samples was carried out using a TA Instruments 2100 series analyzer in the range of 20 ~ 800 °C at a heating rate of 10 °C/min under nitrogen. All samples were dried at 100 °C for 1 hour before the experiment by using TGA.

Ideal absorbed solution theory (IAST) selectivity

The IAST selectivity was calculated as A. L. Myers et.al reported.[Myers, A.L.; Prausnitz, J.M. Thermodynamics of mixed-gas adsorption. *AIChE Journal* **1965**, *11*, 121-127.] The IAST selectivity was derived from the adsorption amount of N_2 and CO_2 measured at 298 K.

Supporting figures

CO_2 adsorption quantity depending on the final heating temperature

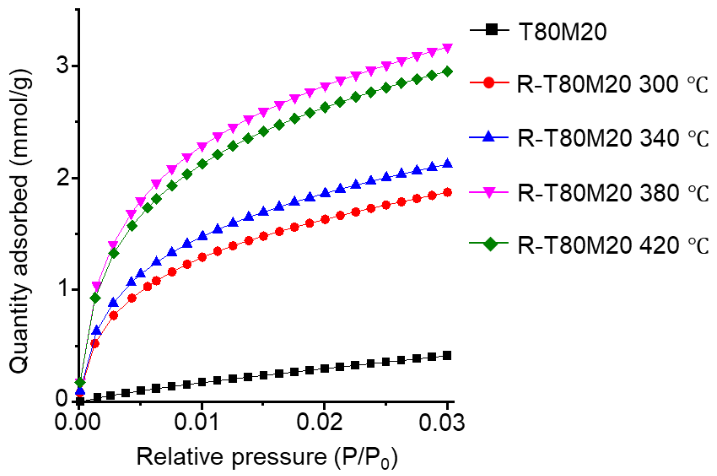

Figure S1. CO_2 adsorption isotherms of T80M20 and R-T80M20s treated at different rearrangement temperatures.

XPS N1s spectrum of co-UNs

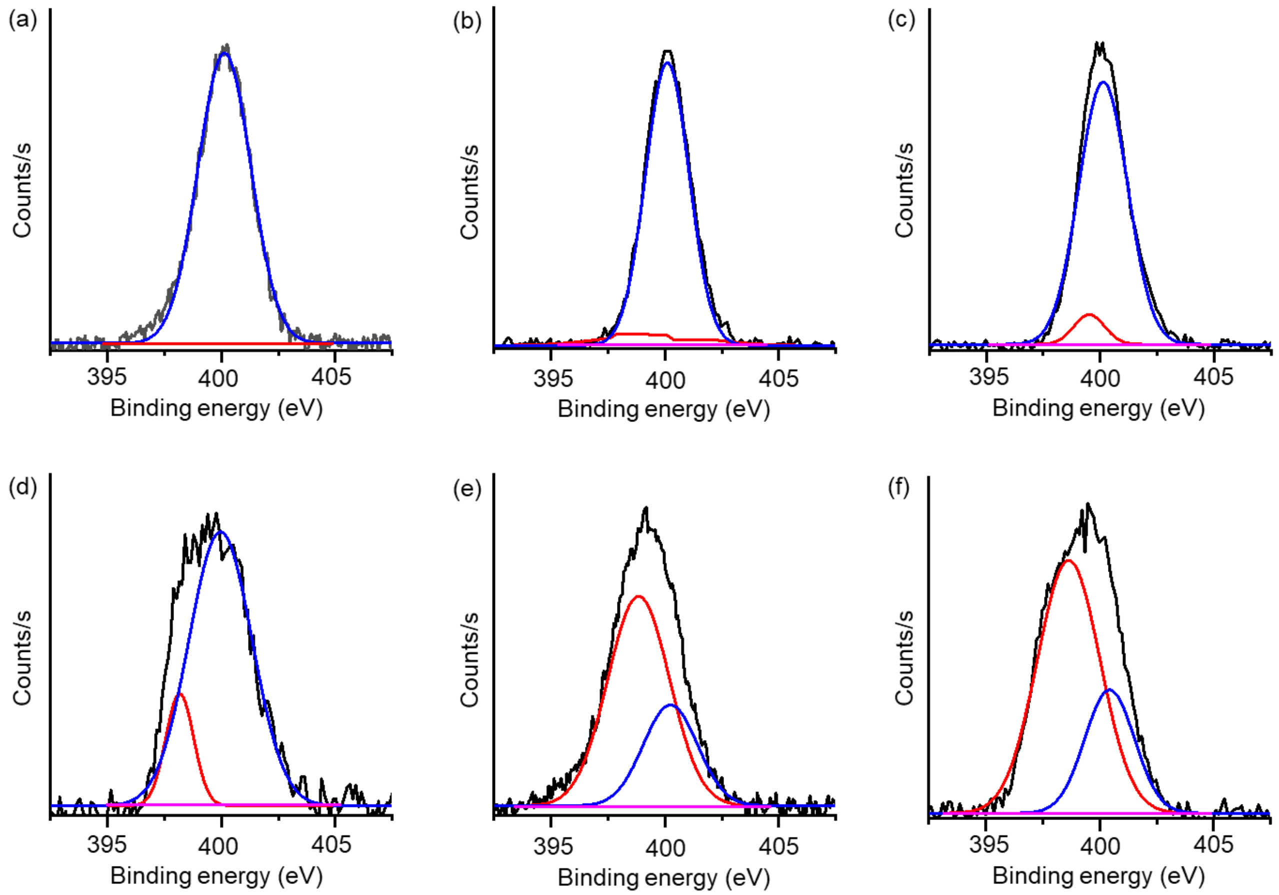

Figure S2. XPS N1s spectra of the co-UNs. (a) T100, (b) T99M1, (c) T80M20, (d) T60M40, (e) T40M60, and (f) M100. Red line correspond to the triazine. Blue line correspond to the urea bond.

FT-IR spectra of co-UNs and co-RUNs

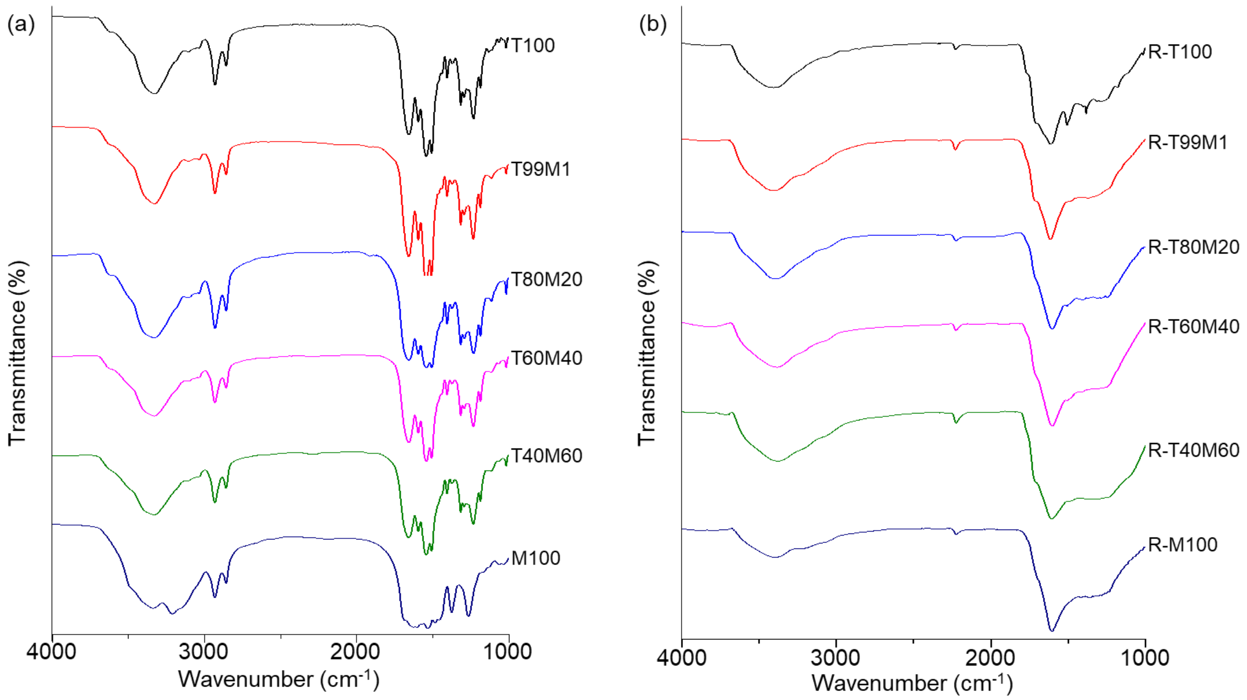

Figure S3. FT-IR spectra of co-UNs and co-RUNs. (a) FT-IR spectra of co-UNs. (b) FT-IR spectra of co-RUNs.

SEM images of co-RUNs

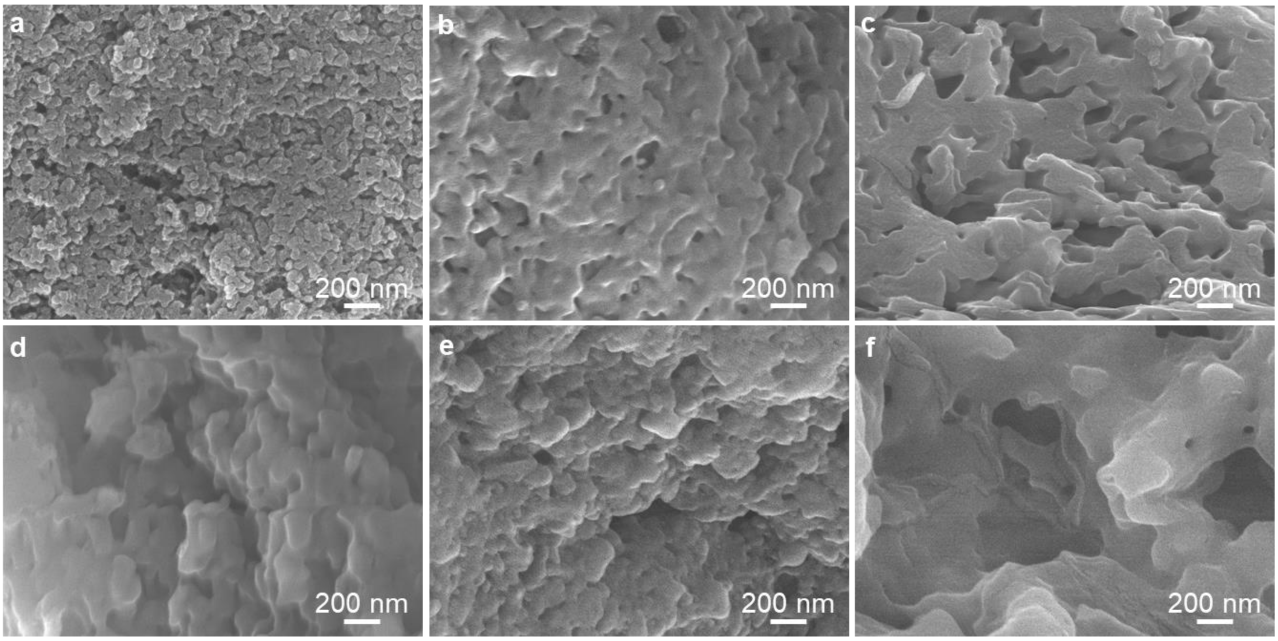

Figure S4. SEM images of co-RUNs. (a) R-T100, (b) R-T99M1, (c) R-T80M20, (d) R-T60M40, (e) R-T40M60, and (f) R-M100.

N_2 and CO_2 adsorption isotherms of co-RUNs collected at 298 K

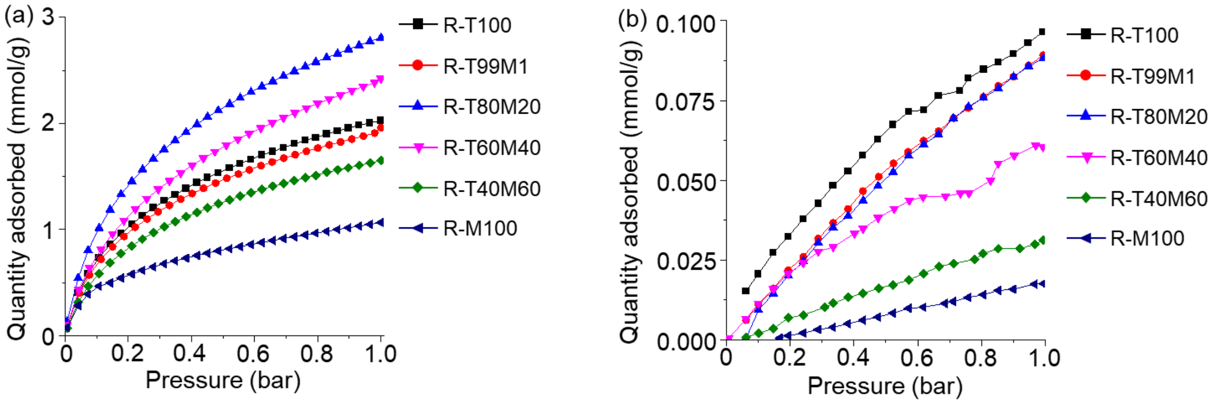

Figure S5. CO_2 and N_2 adsorption isotherms of co-RUNs collected at 298 K. (a) The CO_2 adsorption isotherms of co-RUNs. (b) The N_2 adsorption isotherms of co-RUNs.

CO_2 IAST selectivity of co-RUNs

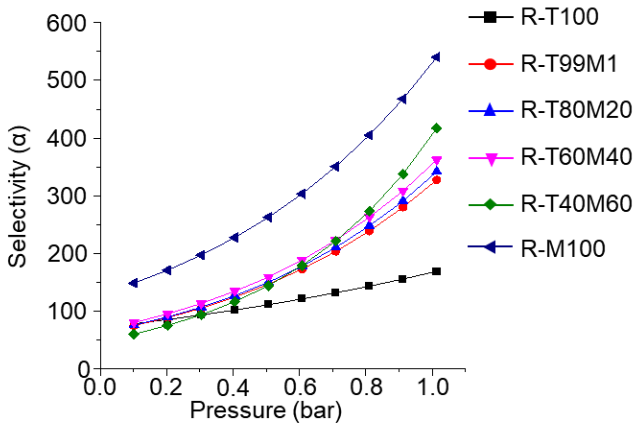

Figure S6. CO_2 IAST selectivity of co-RUNs. The IAST selectivity was derived from the adsorption amount of N_2 and CO_2 measured at 298 K.

CO_2 adsorption-desorption isotherm curve of co-RUNs

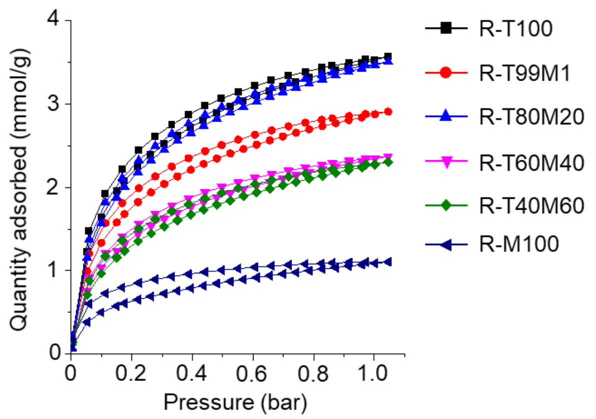

Figure S7. CO_2 adsorption-desorption isotherm curve of co-RUNs. CO_2 adsorption-desorption isotherm curves were collected at 273 K.

CO_2 heat of adsorption of co-RUNs

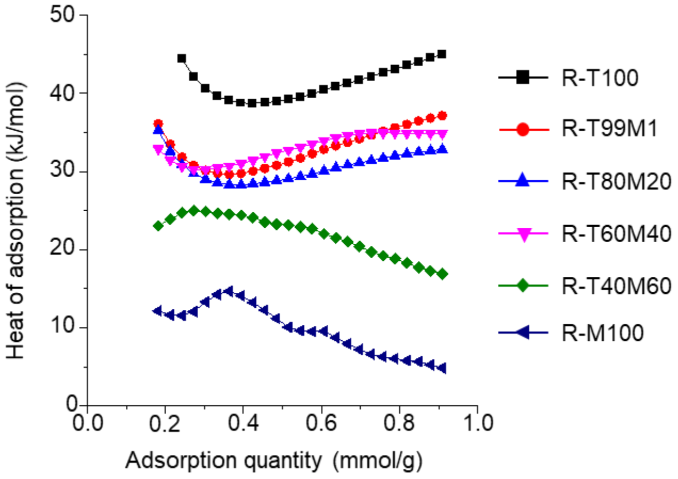

Figure S8. The heat of adsorption of co-RUNs.

Supporting tables

Table S1. Monomer composition of co-UNs.

Sample	TAPM	Melamine	HDI
T100	38.00 g (100.00 mmol)	0.00 g (0.00 mmol)	33.60 g (200.00 mmol)
T99M1	37.70 g (99.00 mmol)	0.13 g (1.00 mmol)	33.50 g (199.50 mmol)
T80M20	30.40 g (80.00 mmol)	2.52 g (20.00 mmol)	31.90 g (190.00 mmol)
T60M40	22.80 g (60.00 mmol)	5.04 g (40.00 mmol)	30.27 g (180.00 mmol)
T40M60	15.22 g (40.00 mmol)	7.57 g (60.00 mmol)	28.59 g (170.00 mmol)
M100	0.00 g (0.00 mmol)	12.61 g (100.00 mmol)	25.23 g (150.00 mmol)

Table S2. Nitrogen functional group ratio of co-UNs. Functional groups ratio was calculated with XPS N1s data.

Sample	Triazine (wt%)	Urea (wt%)
T100	0	100
T99M1	4	96
T80M20	7	93
T60M40	15	85
T40M60	59	41
M100	72	28

Table S3. Elemental ratio of co-UNs and co-RUNs. Elemental ratio was derived from elemental analysis.

Sample	Carbon (wt%)	Nitrogen (wt%)	Oxygen (wt%)	Hydrogen (wt%)
T100	68.4	15.0	10.3	6.3
T99M1	68.4	15.9	9.4	6.3
T80M20	67.5	15.9	10.3	6.2
T60M40	63.7	19.7	10.3	6.2
T40M60	61.9	20.6	12.2	6.1
M100	49.7	32.8	11.2	5.9
R-T100	64.9	11.6	20.3	3.2
R-T99M1	64.7	11.6	20.3	3.4
R-T80M20	66.0	12.6	18.4	2.9
R-T60M40	66.2	14.6	16.6	2.6
R-T40M60	66.3	17.3	14.4	1.9
R-M100	52.2	34.5	11.8	1.5

Table S4. Estimated monomer weight percent of co-UNs.

Sample	TAPM (wt%)	Melamine (wt%)	HDI (wt%)
T100	53.0	0.0	47.0
T99M1	52.7	0.2	47.1
T80M20	46.9	3.9	49.2
T60M40	39.5	8.6	51.9
T40M60	29.4	14.7	55.9
M100	28.9	14.8	56.3

Table S5. BET surface area and t-plot micropore surface area of co-RUNs.

Sample	BET surface area (m^2/g)	t-plot micropore surface area (m^2/g)
R-T100	583	408
R-T99M1	521	357
R-T80M20	523	449
R-T60M40	336	314
R-T40M60	206	165
R-M100	181	151
